# Gastric Glomus Tumor: A Rare Cause of Upper Gastrointestinal Bleeding

**DOI:** 10.1155/2015/193684

**Published:** 2015-12-01

**Authors:** Yoshinori Handa, Mikihiro Kano, Mayumi Kaneko, Naoki Hirabayashi

**Affiliations:** ^1^Department of Surgery, Hiroshima City Asa Hospital, 2-1-1 Kabeminami, Asakita-ku, Hiroshima 731-0293, Japan; ^2^Department of Pathology, Hiroshima City Asa Hospital, 2-1-1 Kabeminami, Asakita-ku, Hiroshima 731-0293, Japan

## Abstract

A 24-year-old woman was referred to our department because of melena. These symptoms combined with severe anemia prompted us to perform an emergency upper endoscopy, which showed bleeding from an ulcerated 30 mm submucosal tumor in the gastric antrum. A computed tomography scan revealed a homogeneously enhanced mass, and endoscopic ultrasonography identified a well-demarcated mass in the third and fourth layers of the gastric wall. Because analysis of the possible medical causes remained inconclusive and the risk of rebleeding, laparoscopy-assisted gastric wedge resection was performed after administration of 10 units of red cell concentrate. Histological and immunohistological analysis revealed the tumor to be a gastric glomus tumor. Gastric submucosal tumors remain challenging to diagnose preoperatively as they show a variety of radiologic and clinicopathologic features and are associated with the risk of bleeding upon biopsy, as is indicated in the guidelines for gastric submucosal tumors. Gastric glomus tumors characteristically present with exsanguinating gastrointestinal hemorrhaging that often requires blood transfusion. Additionally, gastric submucosal tumors typically occur in elderly patients; however, this case involved a young patient who was 24 years old. Here, we describe this case in order to identify features that may aid in early differentiation of gastric submucosal tumors.

## 1. Introduction

Glomus tumors (GTs) are benign lesions originating from modified cells of the glomus body that are responsible for regulation of arteriolar blood flow. Although these tumors usually occur in peripheral soft tissue [1], in the gastrointestinal tract, they are most commonly found in the stomach. We encountered a case of a gastric GT that was characteristic in its clinical course, presenting with exsanguinating gastrointestinal hemorrhage and patient at the age of 24 years. Taken together with the findings in previous reports, the characteristics observed here may be useful for diagnosing gastric submucosal tumors (SMTs).

## 2. Case Presentation

A 24-year-old woman with no previous history of illness presented with vomiting andmelena to her local primary clinic. Tachycardia was noted as a characteristic physical abnormality, and her hemoglobin level was extremely low (6.3 g/dL). The patient was transferred to our hospital and was subjected to further testing along with transfusion of 10 units of red cell concentrate. An emergency upper gastrointestinal endoscopy revealed active bleeding from an ulcer on the surface of an elevated lesion located in the lower portion of the stomach, along the greater curvature. The bleeding was successfully controlled using local sclerosis therapy.

**Figure 1:**
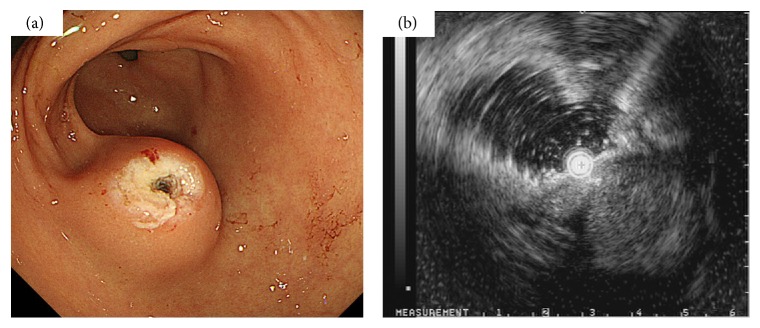
Upper gastrointestinal endoscopy and endoscopic ultrasonography findings. The image shows a well-circumscribed elevated mass, measuring 30 × 30 mm, with normal overlying mucosa in the anterior wall of the gastric antrum (a). The image shows a well-demarcated mass in the third and fourth layers of the gastric wall with a hypoechoic pattern (b).

**Figure 2:**
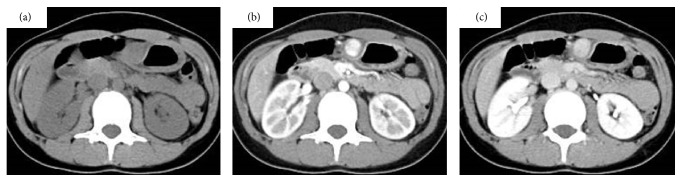
Computed tomography findings. Abdominal computed tomography revealed a well demarcated, ovoid mass at the antrum on (a) unenhanced, (b) arterial phase, and (c) delayed phase scans.

Two days later, the patient underwent another upper gastrointestinal endoscopy, which revealed a 30 mm, well-circumscribed, soft SMT (Figure 1(a)). The gastric mucosa covering the SMT showed a small ulcer, without signs of bleeding. We did not biopsy this mass because of the probability of rebleeding. Endoscopic ultrasonography (EUS) showed a well-demarcated mass in the third and fourth layers of the gastric wall, with a hypoechoic pattern (Figure 1(b)). A computed tomography (CT) scan of the abdomen revealed a mass with dense homogeneous enhancement in the stomach wall. This scan suggested the presence of a lesion with abundant blood supply and an intact overlying mucosa using early- and delayed-phase contrast-enhanced CT, respectively (Figure 2). Because the analysis to identify the possible medical causes of the lesion remained inconclusive and because of the risk of rebleeding, the patient was referred for an elective laparoscopy-assisted surgical procedure, according to the Japanese treatment guidelines for gastric SMTs [2].

During the operation, a tumor, approximately 30 mm in diameter, was found in the antral wall of the stomach. The tumor had not invaded the adjacent organs and was successfully removed via gastric wedge resection. The patient had an uneventful postoperative course and was discharged six days after surgery.

**Figure 3:**
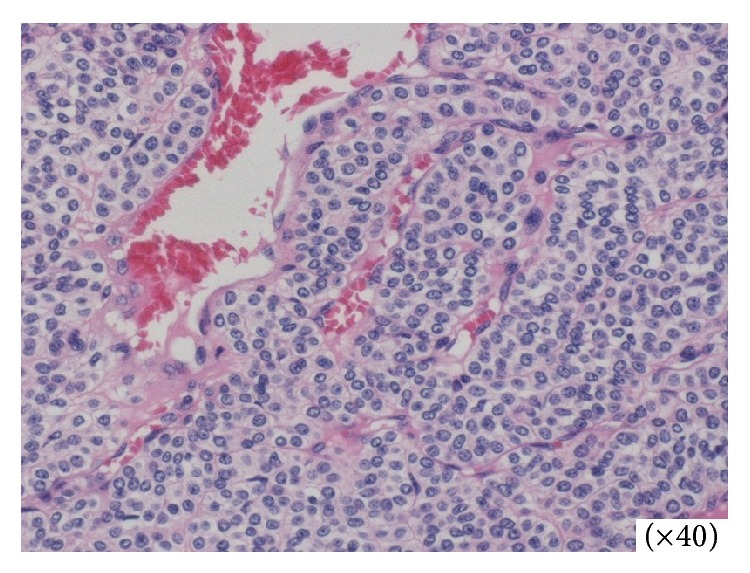
Histological findings. Microscopic examination shows numerous dilated, thin-walled vascular spaces surrounded by uniform glomus cells (hematoxylin and eosin staining; magnification, ×40).

**Figure 4:**
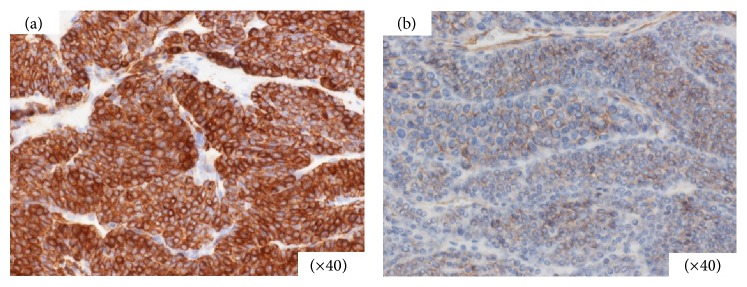
Immunohistological findings. Tumor cells are positive for smooth muscle actin (a) and collage type IV (b) (magnification, ×40).

Histologically, the tumor was composed of atypical glomus cell nests surrounding capillaries (Figure 3). Immunohistochemical staining revealed the tumor cells to be positive for vimentin, smooth muscle actin, and collagen type IV (Figure 4). Staining for CD34, synaptophysin, and chromogranin A revealed that they were not expressed in these tumor cells. Taken together, our analysis led to diagnosis of this lesion as a gastric GT.

The tumor was completely resected and was determined not to be severely malignant based on the detection of few cells undergoing mitosis and the observation of few atypical cells. We therefore chose not to provide adjuvant therapy while continuing to monitor the patient through follow-up. Ten months after the operation, the postoperative course remains uneventful without signs of relapse.

## 3. Discussion

GTs are rare benign mesenchymal tumors arising from the glomus body. This type of tumor was first reported by De Busscher in 1948 [3], and less than 200 reports have been published in the English literature since then. Previous reports suggest that gastric GTs account for approximately 2% of all benign gastric tumors and that they are slightly more frequent in women, with a 2 : 3 male-to-female ratio [4].

Gastric SMTs vary in malignant potential from benign to highly malignant and vary in size from a small nodule to a large mass, such as gastrointestinal stromal tumors (GISTs), heterotopic pancreas, hemangiomas, carcinoid tumors, and neurilemmomas. Additionally, GTs may present as a solitary, hypervascular SMT, as in our case. Among these tumor types, GISTs are the most common ones and are often malignant, so the main diagnostic strategy when examining such a gastric SMT is to differentiate it from GISTs. An ideal course of action would be to perform a preoperative biopsy, but this is often difficult owing to the potential for rebleeding, and guidelines for gastric SMTs recommend operation without a biopsy when patients have symptoms [2]. Radiological and clinicopathologic characteristics can be used to help determine the differential diagnosis [5]. The radiological features of GISTs are diverse; namely, on examination with plain CT, GISTs exhibit low density, show strong enhancement on the arterial-phase images, and do not exhibit prolonged enhancement on delayed-phase images. On EUS, GISTs usually appear as heterogeneous tumors between the submucosal and muscularis propria layers [6]. On the other hand, GTs are reported to show different features from those described for GISTs. On examination with CT, GTs manifest as well-circumscribed submucosal masses with homogeneous density on unenhanced images and show strong enhancement on arterial-phase images as well as persistent enhancement on delayed-phase images, as was observed in our case [7]. On examination with EUS, GTs usually show well-circumscribed hypoechoic masses located in the third and/or fourth layer of the gastric wall [8] although heterogeneous echogenicity, caused by hemorrhage or calcification, may occur.

Patients with gastric SMTs usually present with epigastric pain and distress, having been reported in over 50% of patients. Gastrointestinal bleeding and ulcer-like symptoms are occasionally observed and have been reported in 5% of patients with gastric SMTs. However, exsanguinating gastrointestinal hemorrhage from a SMT is very rare. Gastric GTs are thought to be highly vascularized because this tumor arises from cells of the arteriovenous anastomosis plexus and, in several reports, gastric GTs have led to exsanguinating gastrointestinal hemorrhaging requiring a blood transfusion [9–11].

Additionally, gastric SMTs such as GISTs generally occur in elderly patients, especially in patients above the age of 60; by comparison, this case involved a 24-year-old patient. Although the scarcity of reports may preclude a predictive conclusion, gastric GTs have been reported to occur at younger ages than GISTs, with a median age of 45 years (range, 28–79 years) [12]. Gastric GT should be included as a differential diagnosis if a solitary, hypervascular SMT is detected in the stomach, especially when it presents with exsanguinating gastrointestinal hemorrhaging and occurs in a young patient.

We confirmed that gastrointestinal GTs are histologically and immunophenotypically comparable with the GTs found in peripheral soft tissues, as previously reported [13, 14]. Histologically, the tumor was mainly located in the muscularis of the stomach and was composed of small, uniform, rounded cells surrounding capillaries with diffuse sheet distributions, but without nuclear pleomorphism or mitotic figures. The tumor was positive for *α*-smooth muscle actin and vimentin but negative for CD34 and KIT. These immunohistological features may help to distinguish GTs from other histologically similar tumors.

In general, gastric GTs have a good prognosis; however, several authors have reported that gastric GTs can metastasize hematogenously to the liver, lungs, and brain [15–17], and a tumor diameter of greater than 5 cm might be an indicator of risk [18]. Although gastric GTs are usually small with a median size ranging from 2 cm to 3 cm, as was observed in our case, careful follow-up is recommended.
